# Reply to Dravet, C. Different Outcomes of Acute Encephalopathy after Status Epilepticus in Patients with Dravet Syndrome. How to Avoid Them? Comment on “De Liso et al. Fatal Status Epilepticus in Dravet Syndrome. *Brain Sci*. 2020, *10*, 889”

**DOI:** 10.3390/brainsci11060811

**Published:** 2021-06-18

**Authors:** Paola De Liso, Virginia Pironi, Massimo Mastrangelo, Domenica Battaglia, Dana Craiu, Marina Trivisano, Nicola Specchio, Rima Nabbout, Federico Vigevano

**Affiliations:** 1 Department of Neuroscience, Bambino Gesù Children’s Hospital, IRCCS, Full Member of European Reference Network EpiCARE, 00165 Rome, Italy; paola.deliso@opbg.net (P.D.L.); virginia.pironi@gmail.com (V.P.); marina.trivisano@opbg.net (M.T.); nicola.specchio@opbg.net (N.S.); 2 Center for Rare Diseases and Birth Defects, Department of Woman and Child Health, Institute of Pediatrics, Policlinico Universitario Gemelli Foundation, Catholic University of Rome, 00168 Rome, Italy; 3 Paediatric Neurology Unit, V. Buzzi Hospital, A.O. ICP, 20019 Milan, Italy; massimo.mastrangelo@asst-fbf-sacco.it; 4 Department of Child Neurology and Psychiatry, Policlinico Universitario Gemelli Foundation, Catholic University of Rome, 00153 Rome, Italy; domenicaimmacolata.battaglia@policlinicogemelli.it; 5 Department of Neurology, Paediatric Neurology, Psychiatry, Neurosurgery, “Carol Davila” University of Medicine of Bucharest, Full Member of European Reference Network EpiCARE, 050474 Bucharest, Romania; dcraiu@yahoo.com; 6 Centre for Rare Epilepsies, Department of Paediatric Neurology, Necker-Enfants Malades Hospital, Imagine Institute, INSERMU1163, Paris Descartes University, Full Member of European Reference Network EpiCARE, 75006 Paris, France; rimanabbout@yahoo.com

It has been an honor for us to receive a comment on our article “Fatal Status Epilepticus in Dravet Syndrome” [1] from Charlotte Dravet, the epileptologist who described this syndrome. We thank Prof. Dravet, and we agree with her comments.

We are aware that acute encephalopathy after febrile epileptic status might have a variable outcome; there are indeed some patients who overcome this condition without sequelae, or with significant neurological consequences, and patients who unfortunately die. We pointed out these aspects in our article.

There were two goals of our report: the first was trying to identify the factors that might predict the different outcomes in case of febrile Status Epilepticus (SE) and acute encephalopathy after febrile epileptic status in Dravet Syndrome (DS). The second goal was to alert epileptologists and families to the importance of specific recommendations for the treatment of prolonged seizures, cluster of seizures, and SE in DS.

We identified very generic risk factors, and the cases reported by Prof. Dravet confirm how difficult it is to predict the outcome of acute encephalopathy after febrile epileptic status; two sisters with the same *SCN1A* genetic variant experienced an acute encephalopathy after febrile epileptic status: one died and the other survived.

We must therefore conclude that the risk of severe acute encephalopathy after febrile epileptic status actually exists for all patients with DS, and at all ages.

We endorse the proposal of Prof. Dravet to produce specific recommendations on how to treat prolonged or clustered seizures, as well as fever in DS patients, in order to reduce the incidence of acute encephalopathy after febrile epileptic status.

## References

[1] De Liso P., Pironi V., Mastrangelo M., Battaglia D., Craiu D., Trivisano M., Specchio N., Nabbout R., Vigevano F. (2020). Fatal Status Epilepticus in Dravet Syndrome. Brain Sci..

